# Trends and Challenges: A 15-Year Review of Imaging and Radiation Oncology Core Anthropomorphic Phantom Audits for Proton Therapy

**DOI:** 10.1016/j.ijpt.2025.101297

**Published:** 2026-01-02

**Authors:** Lian Duan, Hunter S. Mehrens, Stephen F. Kry, Jessica R.L. Lowenstein, Nadia Hernandez, Lucas B. Acuna Scafati, Paige A. Taylor

**Affiliations:** 1 Department of Radiation Physics, The University of Texas MD Anderson Cancer Center, Houston, TX, USA; 2 Graduate School of Biomedical Sciences, The University of Texas MD Anderson Cancer Center UTHealth Houston, Houston, TX, USA

**Keywords:** Anthropomorphic phantom, Dosimetry, Quality assurance, Proton therapy, Audit

## Abstract

**Purpose:**

This study aims to analyze the trends in institutional performance on Imaging and Radiation Oncology Core (IROC) proton phantoms and to investigate the impact of treatment parameters on proton therapy delivery accuracy.

**Materials and Methods:**

We analyzed 402 IROC anthropomorphic phantom audits performed by 57 institutions worldwide between 2009 and 2024, including brain, head and neck (H&N), spine, prostate, lung, and liver phantoms. Dosimetric assessments included thermoluminescent dosimeters (TLDs) for dose comparison and gamma analysis of radiochromic film. The lung and liver phantoms were irradiated with simulated motion. Univariate analysis was performed to evaluate the relationships between treatment parameters and phantom outcomes (pass/fail, TLD-to-treatment planning systems (TPS) ratio, and gamma passing rate). Linear regression was used to analyze the long-term trends in the institutional delivery accuracy across the 6 phantoms.

**Results:**

The inter-institutional dose variation in the target was approximately 3% across all phantoms. Of the participating institutions, 66.7% failed at least one IROC proton phantom. Pass rates were 97% for brain, 91% for H&N, 89% for spine, 76% for prostate, 63% for lung, and 57% for liver phantoms. Phantoms incorporating motion exhibited the poorest performance, particularly in gamma analysis, with 33 (*N* = 91) lung and 33 (*N* = 76) liver irradiations showing gamma values below 85%. No linear temporal trends were observed in the overall passing rates of the six phantoms, while TLD results improved for brain, liver, spine, and prostate phantoms (*P* < .05). Machine type was correlated with pass rates for H&N and liver phantoms (*P* < .05).

**Conclusion:**

Overall institutional phantom performance has not significantly improved over time. In particular, the proton lung and liver phantoms continuously demonstrate suboptimal results, likely due to complex geometries and motion management challenges. These findings underscore the need for careful selection of treatment parameters and optimization of motion management strategies. The IROC phantom program remains crucial for characterizing proton therapy systems and identifying clinically significant errors.

## Introduction

Over the past 2 decades, the number of proton therapy centers worldwide has grown substantially, with more than 120 centers currently operational and approximately ten more either under construction or in planning.^1^ The increasing adoption of proton therapy for cancer treatment underscores the critical need for institutions to maintain high-quality care.^2^ The American Association of Physicists in Medicine Task Group- 24 report^3^ and Task Group-185^4^ highly recommended independent dosimetry audits using separate equipment in addition to the institution’s internal Quality Assurance (QA) program. The Global Clinical Trials RTQA Harmonization Group (GHG) highlightedthe importance of RTQA in large multicenter clinical trials for reducing differences, increasing the power of trials, delivering consistency in reporting of trial quality factors, and facilitating effective multinational trials and data analysis.^5^ To ensure the delivery of consistent and accurate clinical treatments, proton therapy centers that plan to participate in National Cancer Institute-funded clinical trials must complete an approval and credentialing process through the Imaging and Radiation Oncology Core (IROC) QA Center.^6^, ^7^

A crucial part of the credentialing process is the IROC anthropomorphic phantom audit. These phantoms serve as comprehensive end-to-end tests of a proton center’s ability to accurately deliver prescribed doses to clinically relevant target volumes while sparing nearby organs at risk (OARs). The phantom program involves sending the phantom to an institution, where clinicians and/or physicians perform CT simulation, treatment planning, and dose delivery following their clinical protocol; the irradiated phantom is then returned to the IROC QA center for analysis. IROC currently utilizes six anthropomorphic proton phantoms designed to simulate different anatomical sites, including the brain, head and neck (H&N),^8^ spine,^9^ prostate,^10^ lung,^11^, ^12^ and liver.^13^, ^14^

We previously reported an overall passing rate of 79% for 5 proton phantoms irradiated before 2015, among which the liver phantom had the lowest pass rate.^10^ Similar results for the liver phantom were found in a second analysis from 2015 to 2022, despite significant advancements made during that time period in proton treatment planning and delivery system technologies,^2^ including the widespread adoption of pencil beam scanning (PBS),^15^ improvements in proton treatment planning systems (TPS), more sophisticated optimization algorithms,^16^ the incorporation of cone-beam computed tomography image-guided radiation therapy,^17^ and increased domain expertise within the proton therapy community.^18^ To further explore how these factors affect passing rates for the different types of phantoms and to look for trends that might help identify barriers to success, this study provides a comprehensive analysis of all proton phantoms irradiated between 2009 and 2024 as part of IROC's dosimetry audit program. The study includes 57 proton therapy centers and 402 phantom irradiations. The results and discussion provide unique insight into quality management in proton therapy delivery.

## Materials and methods

### IROC phantoms

The IROC QA center employs 6 anthropomorphic phantoms for proton therapy site approval and credentialing. These phantoms represent different clinical sites: brain, H&N, spine, prostate, lung, and liver. All phantom materials are chosen to have tissue-equivalent relative linear stopping power (RLSP), ensuring proton interactions comparable to those in human tissue.^19^

**Figure 1:**
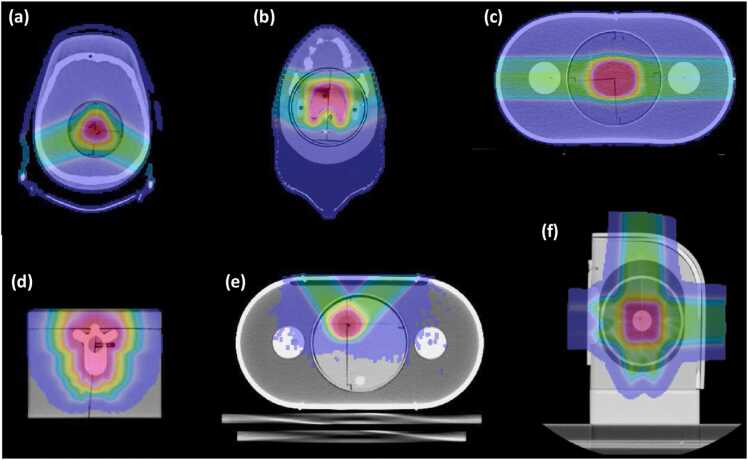
Axial CT simulation slices and corresponding dose maps for the IROC anthropomorphic proton phantoms across various anatomical sites: Brain (a), H&N (b), Prostate (c), Spine (d), Liver (e), and Lung (f). Radiochromic films are visible as orthogonal dark lines at the planning target volume (PTV), and TLD capsules are present within the PTV and organs at risk (OARs). *Note: These examples are for illustration only and are not intended as standard reference plans.*

The proton brain phantom^10^ is constructed from RANDO water-equivalent plastic (Phantom Laboratory, Salem, NY) and contains an embedded human skull. As shown in Figure 1a, the imaging insert includes a 2-cm diameter spherical target simulating a meningioma that is visible on both CT and MRI. The dosimetry insert, made of solid polyethylene, holds radiochromic ﬁlm (GAFChromic EBT3 film, Ashland Inc, Covington, Kentucky) in the coronal and sagittal planes. Two thermoluminescent dosimeter (TLD) capsules are embedded in the target (1 anterior and 1 posterior) to measure the delivered dose.

The proton H&N phantom^8^ (Figure 1b) is made of Alderson water-equivalent plastic (Phantom Laboratory, Salem, New York) and includes an embedded human skull, along with open oral and sinus cavity channels to mimic airways. A cylindrical insert contains a horseshoe-shaped target that partially wraps around a surrogate spinal cord and is placed at the center of the insert. This insert incorporates 3 critical OAR structures: the spinal cord and two parotid glands (positioned laterally). Radiochromic film is inserted in the axial and sagittal planes to measure dose in cross-section, and TLD capsules are placed inside each critical structure—1 in each parotid gland and 2 in the spinal cord and target (at superior and inferior positions of each).

The proton prostate phantom^10^ (Figure 1c) consists of a water-fillable polyvinyl chloride outer shell, 2 femoral heads made of polybutylene terephthalate plastic, and separate imaging and dosimetry inserts. The imaging insert is water-filled for CT scanning and contains a 5 cm diameter spherical target representing the prostate, along with critical structures representing the bladder and rectum. The dosimetry insert is solid polystyrene and holds radiochromic film in the coronal and sagittal planes. Two TLD capsules are embedded within the prostate target (one on the left side and one on the right side), and an additional 2 TLD capsules are placed in each of the femoral head structures to assess bone dose.

The proton spine phantom has existed in 2 versions. Prior to 2015, irradiations used a pig spine phantom, which was a solid phantom containing excised pig vertebral bones.^10^ In 2015, a new pediatric spine phantom was introduced,^9^ featuring a pediatric-sized spine with 12 real vertebral bodies, intervertebral cartilage discs, and a spinal cord analog, all embedded in soft tissue-equivalent material (Figure 1d). The cartilage discs are made of Techtron HPV bearing grade (Quadrant EPP, Lenzburg, Switzerland), and the surrounding soft tissue is Solid Water (Gammex, Middleton, Wisconsin). In both spine phantoms, radiochromic film is placed in the coronal and mid-sagittal planes to capture the planar dose distribution. Two TLD capsules are placed within the spinal canal (one in the upper right region and one in the lower left region of the canal) to measure point doses.

The proton lung phantom^12^ (Figure 1f) represents the left side of the thorax, including lung, soft tissue, and ribs, constructed largely from Solid Water plastic (Gammex, Middleton, WI). The left lung is simulated with a block of compressed cork that contains a movable low-density balsa wood insert for dosimetry. The rib cage is represented by high-density Techtron HPV plastic (Quadrant EPP, Lenzburg, Switzerland), and the surrounding soft tissue is Solid Water. The lung insert features an elongated cylindrical target (∼3 cm diameter, 5 cm length) in which radiochromic films are placed in all three orthogonal planes (axial, sagittal, and coronal). Two TLD capsules are also embedded in this target volume. The respiratory motion of the lung was simulated using a dosimetry insert that moves independently of the outer shell. The insert's repeated linear motion was controlled by a programmed translational stage to have an amplitude of 2 cm and a respiration rate of 10 cycles per minute. This setup allows institutions to test various motion management protocols, including breath-hold, respiratory gating, an internal target volume (ITV) approach, and treatment under static (no-motion) conditions.

The proton liver phantom^13^ (Figure 1e) consists of a water-fillable polyvinyl chloride shell enclosing an insert with 2 separate targets. These 2 targets are made of solid water and are positioned non-coplanarly (at different 3-dimensional orientations within the liver volume). PTV1 (target 1) is an oblong spheroidal target approximately 2 cm in size located in the superior left portion of the insert, and PTV2 (target 2) is a roughly 3 cm spherical target located in the inferior right portion. Each target contains 2 radiochromic films in the sagittal and coronal planes and 2 TLD capsules. In addition, the liver phantom has 2 lateral structures made of polybutylene terephthalate plastic that represent normal liver tissue on the left and right sides of the phantom. The liver phantoms can be mounted on an acrylic motion platform to simulate respiratory motion during irradiation. This platform produces a sinusoidal motion with an amplitude of 1 cm, allowing for the simulation and testing of an institution’s motion management protocols.

### Dataset

Between 2009 and 2024, a total of 402 phantom irradiations were delivered across 57 proton therapy institutions and subsequently analyzed by IROC. This dataset includes all submitted phantom irradiations, including initial failures and subsequent repeated measurements. In the IROC anthropomorphic phantom audit program, phantoms are shipped to participating institutions, where they are instructed to follow their standard clinical workflows, delivering a treatment plan such that the target(s) is covered by at least the 95% isodose line. The irradiated phantoms are then returned to IROC for independent analysis. Detailed criteria for each phantom type are provided in Table 1.

**Table 1:** Passing criteria, numbers of irradiation, overall pass number and percentages, and failure types for 6 anthropomorphic proton phantoms.

Phantom type	Brain	H&N	Spine	Prostate	Lung	Liver
TLD criteria	0.95-1.05	0.93-1.07	0.95-1.05	0.93-1.07	0.92-1.05	0.93-1.07
Gamma criteria	5%/3 mm	7%/4 mm	5%/5 mm	7%/4 mm	7%/5 mm	7%/4 mm
Gamma threshold	85%	85%	85%	85%	85% (80%*)	85%
Total number	58	65	37	75	91	76
Passing rate	56 (97%)	59 (91%)	33 (89%)	57 (76%)	57 (63%)	43 (57%)
TLD failed	1	4	-	1	9	7
Gamma failed	2	5	4	18	33	33
TLD and gamma failed	1	2	-	1	8	7

The TLD criteria refers to the worst agreement on any individual (double-loaded) TLD.

* For the lung phantom, each individual film plane must achieve a minimum pass rate of 80% of pixels, while the overall average pass rate across all 3 film planes must exceed 85%.

### Statistical analysis

To investigate the impact of various treatment parameters on audit outcomes (pass/fail), we conducted Chi-square tests to examine the relationships between outcomes and the following treatment parameters: machine type, delivery technique, TPS, dose calculation algorithms, and motion management protocols. Pairwise t-tests were performed to determine whether there were statistically significant differences in TLD-to-TPS dose ratios or gamma passing rates between different parameter constituents. To analyze trends in institutional performance over time, logistic regression was employed to evaluate the relationship between the time of irradiation and the binary audit outcomes. Similarly, linear regression was used to assess any temporal changes in TLD ratios and gamma passing rates for each phantom.

## Results

**Figure 2:**
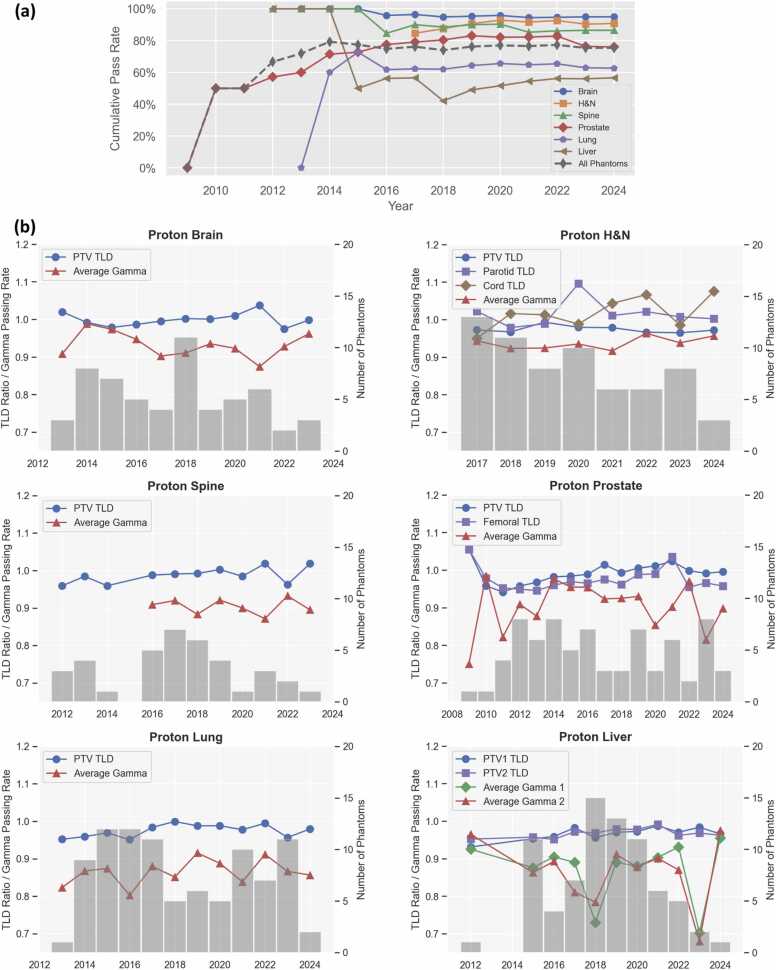
**(a)** Cumulative pass rate over time for each proton phantom. **(b)** Average TLD ratios and gamma passing rate values by year for each proton phantom. The number of irradiated phantoms is indicated in the bar graphs.

Among 57 institutions that participated in the IROC proton audit program, 21 unique institutions irradiated all six proton phantoms. Notably, 38 (66.7%) institutions failed at least 1 phantom. A total of 305 (76%) irradiations passed the IROC anthropomorphic phantom audit criteria, while 97 (24%) failed. As detailed in Table 1, the overall passing rates for individual phantoms varied from 57% to 97%. The proton liver phantom had the lowest pass rate, closely followed by the proton lung phantom, whereas the proton brain phantom exhibited the highest overall pass rate. Figure 2a shows that the cumulative pass rate for all six phantoms from 2014 to 2024 fluctuated between 74% and 80%. When examined individually, cumulative passing rates ranged from 94% to 100% for the brain, 85% to 93% for H&N, 85% to 90% for the spine, 73% to 83% for the prostate, 62% to 73% for the lung, and 42% to 58% for the liver phantom. Figure 2b presents the annual average TLD ratios in the planning target volume (PTV) and critical structures, along with gamma passing rate values derived from film analysis for each phantom type. The liver phantom exhibited the poorest performance, with annual average gamma passing rates falling below 80% in 2 separate years. Similarly, the lung phantom showed poor gamma values, ranging from 82.3% to 91.5%. Across all six phantoms, no statistically significant linear trends were observed in the pass/fail outcome over time (*P* > .1). Similarly, no significant improvements were observed between the irradiation time and gamma values for all phantoms or between the irradiation time and TLD ratios for H&N and lung phantoms. However, linear regressions indicated significant improvements in PTV TLD ratios over time for the brain, spine, prostate, and liver phantoms (*P* < .05).

**Figure 3:**
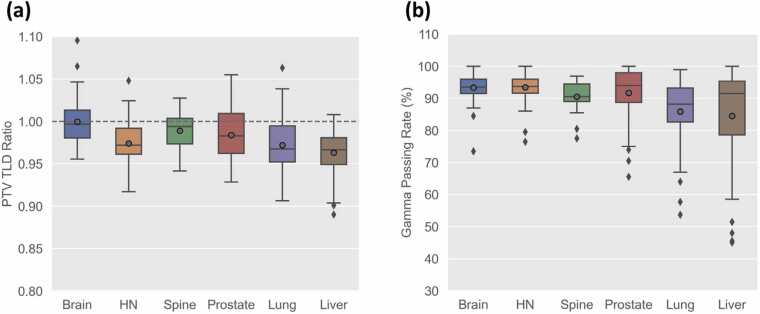
**(a)** PTV TLD-to-TPS dose ratios and **(b)** plane gamma passing rates. The line inside the box represents the median of the data, and the gray circle marker represents the mean value. The edges of the box represents the first quartile (25th percentile) and the third quartile (75th percentile). The whiskers extend to 1.5 times the interquartile range (IQR) from the edges of the box.

Figure 3 illustrates the distribution of PTV TLD ratios and gamma passing rates for each phantom. For 5 phantoms, except the brain phantom, both the mean and median TLD ratios were below 1, indicating that the measured point doses were generally lower than the TPS-calculated doses. The best agreement in the PTV TLD ratio was seen for the brain, for which the ratio was 1.00 and close to the ideal value, followed by the spine and prostate phantoms (0.99). The standard deviations of their PTV TLD ratios across the six phantoms ranged from 0.020 to 0.031. Overall, more heterogenous phantoms such as the lung and prostate showed greater variations across all irradiations, while the relatively homogeneous spine phantom showed the smallest deviation. The highest standard deviations in the average gamma value were observed in the liver phantom (15.0%), followed by the lung (10.3%) and prostate (8.1%) phantoms; all other phantoms showed deviations below 5.0%. All 39 spine phantom irradiations met the PTV TLD criteria, while 1 overdose and 1 underdose case were observed in the brain phantom, and 1 overdose was observed in the prostate phantom. The H&N, lung, and liver phantoms, with average TLD ratios around 0.97, experienced a higher frequency of TLD dosimetry failures. Specifically, the H&N and lung phantoms recorded 4 and 7 underdosed irradiations, respectively, while the liver phantom also exhibited 3 cases of overdosing and 6 cases of underdosing. Overall, 16 of the 22 TLD failures were underdosed, suggesting that the TPSs tend to overestimate the dose delivered to the target in heterogeneous tissue-equivalent media. Of the 97 phantom failures, 95 failed the gamma analysis criteria. Institutions that failed the TLD criteria typically also failed the gamma analysis, as indicated in Table 1. The H&N and the brain phantoms demonstrated the highest mean gamma passing rate (about 93.4%). The prostate and spine phantoms exhibited slightly lower mean gamma passing rates of 90.7% and 90.5%, respectively. In contrast, the lung and liver phantoms—irradiated on a motion platform—showed the poorest gamma passing rates at 86.5% and 85.6%, respectively, with each phantom accounting for 33 gamma failures, the highest number observed.

Table 2 summarizes the demographic data and pass rates for each treatment parameter category. Chi-square tests revealed that machine type was significantly associated with the pass/fail outcomes for the H&N and liver phantoms (*P* < .05). Specifically, lower pass rates were observed with the Mevion system for both H&N and liver phantoms. The overall pass rate for all phantoms was 87% for Hitachi, 79% for Varian, 76% for IBA, and 68% for Mevion. Regarding delivery techniques, 75% used PBS, achieving the highest pass rate of 77%; 9% employed uniform scanning with a 72% pass rate; and 16% used passive scattering with a 71% pass rate. The 2 most commonly used TPSs were Eclipse (37%) and RayStation (45%). With respect to dose calculation algorithms, 38% of cases employed Monte Carlo methods and 62% used pencil beam algorithms; both groups exhibited a pass rate of approximately 75%. Chi-square tests showed no statistically significant association between phantom pass/fail outcomes and delivery technique, TPS, algorithm, or motion management protocol.

**Table 2:** Demographics and pass rates of 6 proton phantoms.

		Proton Brain		Proton H&N		Proton Spine		Proton prostate		Proton Lung		Proton Liver		Total	
Category	Constituents	*N* (%)	Pass rate, %	*N* (%)	Pass rate, %	*N* (%)	Pass rate, %	*N* (%)	Pass rate, %	*N* (%)	Pass rate, %	*N* (%)	Pass rate, %	*N* (%)	Pass rate, %
Machine															
	Hitachi	12 (21%)	100	10 (15%)	100	6 (16%)	83	10 (13%)	70	10 (11%)	70	5 (7%)	100	53 (13%)	87
	IBA	24 (44%)	96	18 (28%)	100	13 (35%)	85	29 (39%)	72	30 (33%)	67	33 (43%)	55	147 (37%)	76
	Mevion	7 (12%)	88	13 (20%)	77	8 (22%)	100	12 (16%)	83	21 (23%)	62	17 (22%)	35	78 (19%)	68
	Varian	9 (16%)	100	18 (28%)	94	8 (22%)	88	16 (21%)	81	23 (25%)	56	16 (21%)	75	90 (22%)	79
Technique															
	PBS	45 (78%)	96	54 (83%)	93	24 (65%)	83	53 (71%)	77	72 (79%)	62	53 (70%)	64	301 (75%)	77
	Passive Scatter	9 (16%)	100	10 (15%)	80	9 (24%)	100	11 (15%)	82	13 (14%)	62	14 (18%)	29	66 (16%)	71
	Uniform Scanning	4 (7%)	100	1 (2%)	100	4 (11%)	100	11 (15%)	64	6 (7%)	67	9 (12%)	56	35 (9%)	72
TPS															
	Eclipse	19 (33%)	100	22 (34%)	96	17 (46%)	88	29 (39%)	79	37 (41%)	51	25 (33%)	52	149 (37%)	74
	RayStation	27 (47%)	93	36 (55%)	92	13 (35%)	85	27 (36%)	74	41 (45%)	71	35 (46%)	57	179 (45%)	77
	XiO	6 (10%)	100	-	-	3 (8%)	100	10 (13%)	70	5 (5%)	60	7 (9%)	57	31 (8%)	74
Algorithm															
	Monte Carlo	19 (32%)	95	26 (40%)	92	11 (30%)	82	23 (31%)	70	47 (52%)	62	27 (36%)	63	153 (38%)	74
	Pencil Beam	39 (67%)	97	39 (60%)	90	26 (70%)	88	52 (69%)	79	44 (48%)	64	49 (64%)	53	249 (62%)	76
Motion															
	Breath Hold									19 (21%)	63	14 (18%)	79	33 (8%)	70
	Gating									6 (7%)	83	5 (7%)	60	11 (3%)	73
	ITV									61 (67%)	59	52 (68%)	50	113 (28%)	55
	Static									5 (5%)	80	5 (7%)	60	10 (2%)	70

The constituents with fewer than 3 samples were excluded in this table.

For the proton brain phantom (detailed in Supplementary Table 1), pairwise t-tests revealed that the XiO TPS produced significantly higher average gamma passing rates than both RayStation (*P* < .05) and Eclipse (*P* < .05). Although the mean TLD ratio was about 1 for all 3 TPSs, the gamma passing rate for RayStation (91.9% ± 5.3%) was lower than those for Eclipse (95.1% ± 3.5%) and XiO (96.7% ± 3.7%). In addition, among the various machine types, Mevion showed the poorest performance in both TLD dose ratios and average gamma passing rates.

Supplementary Table 2 provides a detailed breakdown for the proton H&N phantom. The Hitachi system exhibited superior performance in terms of average gamma passing rate compared to Mevion and Varian, with a significantly smaller mean gamma value of 96.2% (*P* < .05) and a narrower range (7%). Despite the limited sample sizes for some techniques (eg, Passive Scatter, *N* = 10; Uniform Scanning, *N* = 1), the PBS technique achieved favorable outcomes, with a PTV TLD ratio of 0.98 ± 0.02 and an average gamma passing rate of 94.1% ±;3%. Notably, the TLD ratios for the parotid glands and spinal cord showed larger standard deviations and ranges, indicating greater dose variation within these OARs.

For the proton spine phantom (Supplementary Table 3), average PTV TLD ratios did not vary significantly among different treatment parameters, with all values exceeding 0.98. However, overall gamma passing rates in the sagittal plane were significantly lower than those in the coronal plane (*P* < .001), a factor that contributed to all four spine phantom irradiation failures.

Results from the proton prostate phantom (Supplementary Table 4) indicated that the PBS technique yielded superior average TLD dose ratios for both the PTV (1.00 ± 0.03), along with higher gamma passing rates (91.2% ± 8.3%). In addition, the passive scatter technique achieved better PTV and femoral TLD ratios than uniform scanning (*P* < .05). Among the different TPSs, RayStation achieved a better PTV TLD ratio (1.00 ± 0.03) compared to Eclipse (0.98 ± 0.03) and XiO (0.97 ± 0.04), with these differences reaching statistical significance (*P* < .05). Monte Carlo algorithms showed better PTV TLD performance compared to pencil beam (*P* < .001). When comparing pass and fail groups, *t*-tests revealed no significant differences in PTV or femoral TLD dose ratios; however, a significant difference was observed in the average gamma passing rate (*P* < .001).

**Figure 4:**
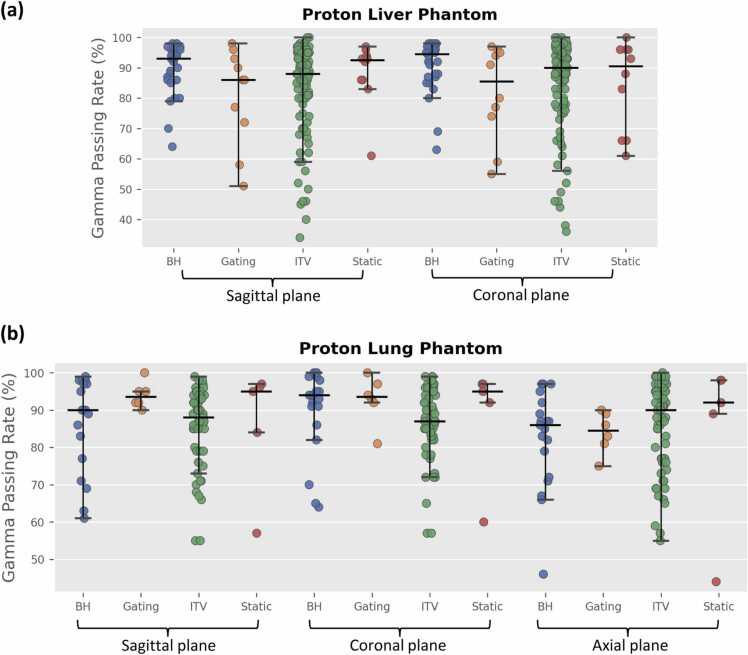
Gamma passing rates for different planes using motion management techniques in proton liver **(a)** and lung phantoms **(b)**. The stack scatter plot displays the distribution of gamma passing rates, while the I-shaped boxplot shows the mean and interquartile range (IQR). The central horizontal line in each box indicates the median, while the box edges denote the first (25th percentile) and third quartiles (75th percentile). Whiskers extend from the box edges to 1.5 times the IQR, capturing the range of the data.

For the proton lung phantom (Supplementary Table 5), no statistically significant differences were found in PTV TLD dose ratios or gamma passing rates when comparing different machine types, delivery techniques, TPS, or algorithms. Statistically significant differences were observed between the pass and fail groups for both average PTV TLD ratios (*P* < .05) and gamma passing rates (*P* < .001). Among motion management techniques, the gating method demonstrated the best performance, whereas the ITV technique yielded the lowest gamma passing rate, with an overall pass rate of 59%. The distribution of gamma passing rates across different motion management strategies is illustrated in Figure 4.

For the proton liver phantom (Supplementary Table 6), the Hitachi system produced better average gamma passing rates for both PTV1 (95.1% ± 1.9%) and PTV2 (90.4% ± 7.6%), followed by the Varian system. The Mevion system exhibited the poorest performance, with mean gamma passing rates below 80% for both PTVs—a difference that was statistically significant. Monte Carlo showed significantly better TLD performance than pencil beam (*P* < .05). No significant differences were observed in PTV TLD dosimetry between different machine types, suggesting that point-dose discrepancies are less pronounced than the differences detected in the planar gamma analysis. However, significant differences were found between delivery techniques (PBS vs uniform scanning and passive scatter vs uniform scanning) and among TPS (RayStation vs. Eclipse and Eclipse vs XiO). In terms of motion management, the ITV technique had the poorest performance with a pass rate of 50%, while the breath-hold technique yielded a higher average gamma passing rates than ITV (*P* < .05). Significant differences in TLD dose ratios and gamma passing rates between pass and fail groups were also observed for both PTVs. Despite the distinct shapes and anatomical locations of the two targets, no statistically significant differences in performance were found between them across the entire dataset.

## Discussion

Independent IROC proton phantom audits have revealed notable performance gaps at many proton therapy centers. A considerable fraction of institutions have not passed an IROC anthropomorphic proton phantom test. Over the past decade of audits, the overall passing rate has fluctuated between approximately 74% and 80%, with no statistically significant improvement in the pass/fail outcome for most phantom types. These findings are consistent with previous reports that demonstrated stagnant pass rates despite technological advancements in proton therapy techniques and equipment.^13^ One possible explanation for this plateau is that, while we are gaining familiarity and competency with techniques, the field is advancing and changing in pace with this, preventing us from truly mastering advanced procedures, including image-guided radiation therapy integration, PBS delivery, TPS dose calculation algorithms, more complex treatment plans, and motion management techniques.

Approximately 5.5% of phantom irradiations failed the TLD criteria. Overall, the average TLD ratios for all phantoms were slightly below 1 (see Figure 3a), implying systematic underdosing relative to the planned dose. This underdosing was less evident in the relatively homogeneous proton brain phantom, which exhibited an average PTV TLD ratio of 1.00, and more pronounced in the proton lung and liver phantoms, where the average PTV TLD ratio was 0.97. Although the differences were not always statistically significant, underdosing was slightly more evident in phantom irradiations using pencil beam algorithms.^12^ Furthermore, the majority of phantom failures occurred due to unmet gamma criteria, and institutions that failed the TLD criteria also typically failed the gamma analysis. This suggests that while many centers can achieve acceptable point doses in the target, they struggle to deliver the planned 2D dose distribution accurately—a challenge that is particularly pronounced in moving phantoms. The mean gamma passing rate was below 85% under the 7%/4 mm gamma criteria, and the standard deviation exceeded 15% across all irradiations for the liver phantom, which contains two non-coplanar targets. These results differ from those of traditional photon treatments on static phantoms; for example, in studies involving the IROC IMRT H&N phantom, most failures (11 out of 16 irradiations) were identified by TLD.^20^ Historical IROC data indicates pass rates of approximately 90% for the IMRT H&N phantom,^21^ 85% for the SRS head phantom,^22^ 89% for SBRT lung, and 84% for SBRT spine phantoms.^11^

OAR dosimetry is inherently more sensitive to delivery errors, a finding consistent with investigations of photon phantoms.^23^ OARs show significantly higher dose variations than the target is their placement in a high-dose gradient region. While the target TLDs are located in a region of uniform high dose (close to 100%), the OARs are situated where the plan attempts to reduce the dose from 100% to near 0% over a very short distance. The dose gradients, along with volume-averaging of the cylindrical powder TLD capsules, result in more dose variation. While TLD agreement is reported for OARs in the proton phantoms, these data are not considered for the pass/fail criteria.

A primary source of range uncertainty in proton therapy remains the accuracy of the CT number to RLSP conversion. This was evident in the high rate of gamma failures in the prostate phantom, where range prediction errors caused discrepancies between the treatment plans and delivery. Previous on-site audits by IROC have evaluated this accuracy by scanning phantoms with known RLSP values at proton centers and compared their clinical curves with the calibration curves collected from all proton centers. Notably, historical data indicates that approximately 19 out of 32 (59%) institutions initially failed to achieve optimal agreement for their CT-to-RLSP conversion curves.^7^ In some cases, clinical curves deviated by more than 15% from the measurement-based criterion, particularly in the low CT number region critical for lung planning. These systematic errors directly degrade performance on anthropomorphic phantoms; however, in instances where institutions corrected their conversion curves and resubmitted plans, the calculated dose distributions typically showed significantly improved agreement with measurements. To address these variations, a standardized consensus guide has been established for generating Hounsfield Look-Up Tables (HLUT) to accurately predict stopping-power ratios.^24^ This approach defines a rigorous 6-step process that combines physical measurements with stoichiometric calculations to ensure robust calibration across institutions, including phantom setup, CT acquisition, CT number extraction, SPR determination, HLUT specification, and HLUT validation.

Another major source of error is the localization of dose in the direction of motion. Motion phantoms—specifically those for lung and liver—consistently showed the lowest pass rates and poorest average gamma values. Respiratory motion introduces significant challenges,^25^ such as the interplay between spot scanning and tumor motion, residual positional uncertainties during gating or breath-hold, and limitations inherent in ITV-based planning. For both the proton lung and liver phantoms, the use of ITV-based motion management resulted in lower pass rates, which is consistent with a previous study.^13^ Although layer and volumetric repainting have been employed by many institutions to mitigate interplay artifacts in PBS, these parameters were not collected for all irradiations, and repainting was not statistically isolated in this study. However, the persistence of lower pass rates suggests that repainting alone could not fully compensate for other errors, particularly those inherent to ITV-based planning. The proton lung phantom often fails because pencil beam-based dose calculations are suboptimal for low-density media and because the interplay between beam scanning and motion can degrade the planned dose distribution.^12^, ^16^ In previous findings,^12^ analytic algorithms were shown to overestimate dose in the target by 7.2%. While we acknowledge that our current dataset did not show a statistically significant difference (*P* > .05), a distinct trend is visible in Supplementary Table 5: Monte Carlo algorithms yielded a higher average passing rate (87.7%) compared to Pencil Beam algorithms (85.3%). Similarly, the complexity of the proton liver phantom—characterized by multiple targets and motion—leads to increased dose discrepancies, as evidenced by notably lower gamma passing rates. These results are similar to those found for traditional photon treatment deliveries on moving phantoms; more than 50% of lung and liver failures were due to a superior-infereior localization error,^26^, ^27^ whereas motion-induced localization errors accounted for only 13% in the static spine phantom.^26^ Analysis of both the lung and liver photon phantoms revealed generally worse agreement for the ITV technique and the best agreement for the tracking technique.

A peer-to-peer intercomparison among 4 German proton centers using PBS found that the systematic deviation in measured absorbed dose between institutions was less than 1%.^28^ However, other audits highlight the potential for significant discrepancies. One independent audit of five European centers found systematic differences of up to 2.7% between the dose measured by the auditor's reference ionization chamber and the dose determined by one center's local reference system.^29^ A multi-center pilot study aimed at developing a postal audit system for scanning proton beams using radiophotoluminescent glass dosimeters estimated a total dosimetry uncertainty of 2.92% after standardizing the reference irradiation conditions.^30^ To ensure the safety of proton therapy and to minimize the dose variability in large-scale multi-institutional trials, there is a strong need for a comprehensive independent audit study involving a larger number of participating institutions.^5^, ^31^ Compared to standard reference measurements, we believe end-to-end tests provide more clinically relevant values, considering the heterogeneities and complexity of IROC’s anthropomorphic phantoms. The IROC phantom program covers the most common clinical sites in clinical trials. The uncertainty in a point dose determination was 1.3% for TLD,^32^ along with the spatial precision of 1 mm the film and densitometer system.^33^ In this study, we reported the inter-institutional variations at a one sigma level of approximately 2%-3% in point dose comparison and 4%-15% in gamma analysis.

IROC’s phantom audits have generated a substantial body of data regarding proton therapy treatment practices.^34^ In this study, we present a comparative analysis of various treatment parameters for proton phantoms, providing valuable feedback for institutions to optimize clinical practices and improve performance on IROC assessments—for instance, by selecting appropriate algorithms or enhancing motion management strategies. Despite being the largest multi-institutional dataset of proton phantom performance, certain subgroups in our study lacked sufficient statistical power to reach definitive conclusions. Given the large number of institutions represented, univariate comparisons alone may not capture the complex interactions present in clinical settings. Nonetheless, this data set accurately reflects current practices and highlights potential deficiencies in clinical workflows related to proton therapy delivery, as well as key factors that may enhance performance. Future work will include collecting additional data and conducting comprehensive multivariate analyses to investigate the relative contributions of various treatment and machine parameters to phantom outcomes within an integrated model.

## Conclusion

Overall, there was no significant improvement in proton phantom performance over time. In particular, the proton liver and lung phantoms consistently exhibited suboptimal results, likely due to the complex geometries, heterogeneities, and challenges associated with motion management. We recommend that institutions carefully select treatment parameters and enhance their motion management strategies. The IROC phantom program plays a critical role in characterizing the complete proton therapy system by identifying and addressing a range of clinically significant errors, ultimately guiding proton centers toward improved accuracy in patient care.

## Funding

This work was supported by Public Health Service Grant CA180803, awarded by the National Cancer Institute, United States Department of Health and Human Services.

## Data availability

The authors will share data upon request to the corresponding author.

## CRediT authorship contribution statement

Lian Duan: Conceptualization, Formal Analysis, Writing. Hunter S. Mehrens: Conceptualization, Formal Analysis, Writing. Stephen F. Kry: Conceptualization Formal Analysis, Writing – review & editing. Jessica R. L. Lowenstein: Data curation, Investigation, Writing – review & editing. Nadia Hernandez: Data curation, Investigation. Lucas B. Acuna Scafati: Data curation, Investigation. Paige A. Taylor: Data curation Conceptualization, Investigation, Formal Analysis, Writing.

## Declaration of Competing Interest

The authors declare that they have no known competing financial interests or personal relationships that could have appeared to influence the work reported in this paper.

## Footnotes

Appendix A Supplementary data associated with this article can be found in the online version at doi:10.1016/j.ijpt.2025.101297.
