# Supplementary material for: Trends and Challenges: A 15-Year Review of Imaging and Radiation Oncology Core Anthropomorphic Phantom Audits for Proton Therapy
Source: Int J Part Ther. 2026 Jan 2;19:101297. doi: 10.1016/j.ijpt.2025.101297 (PMC12825056; doi:10.1016/j.ijpt.2025.101297)
Supplement: Supplementary file 1 — Supplementary material [file mmc1.docx]

**Supplementary Table 1.** Mean Values, Standard Deviations (SD), and Ranges of TLD-to-TPS Dose Ratios and Gamma Passing Rates across Treatment Parameters for the Proton Brain Phantom.

|  |  | Average TLD | | Average Gamma (%) | |
| --- | --- | --- | --- | --- | --- |
|  | **Category** | Mean ± SD | Range | Mean ± SD | Range |
| Machine | Hitachi | 0.99 ± 0.01 | 0.04 | 93.8 ± 4.9 | 13.0 |
|  | IBA | 1.00 ± 0.02 | 0.08 | 94.2 ± 4.3 | 15.5 |
|  | Mevion | 0.93 ± 0.24 | 0.73 | 82.9 ± 20.3 | 61.5 |
|  | Varian | 0.99 ± 0.02 | 0.05 | 94.6 ± 2.4 | 8.5 |
|  | Other | 1.01 ± 0.03 | 0.09 | 91.8 ± 4.3 | 12.0 |
| Technique | PBS | 1.00 ± 0.03 | 0.13 | 92.6 ± 4.8 | 26.5 |
|  | Passive Scatter | 0.93 ± 0.20 | 0.66 | 89.6 ± 19.3 | 64.0 |
|  | Uniform Scanning | 1.00 ± 0.02 | 0.05 | 96.8 ± 3.8 | 8.0 |
| TPS | Eclipse | 1.00 ± 0.02 | 0.08 | 95.1 ± 3.5 | 11.0 |
|  | RayStation | 1.00 ± 0.03 | 0.13 | 91.9 ± 5.3 | 26.0 |
|  | XiO | 1.00 ± 0.02 | 0.05 | 96.7 ± 3.7 | 8.0 |
|  | Other | 0.92 ± 0.25 | 0.70 | 83.2 ± 21.2 | 63.5 |
| Algorithm | Monte Carlo | 1.00 ± 0.03 | 0.10 | 91.6 ± 5.5 | 25.0 |
|  | Pencil Beam | 0.98 ± 0.10 | 0.70 | 92.8 ± 10.1 | 64.0 |
| Pass / Fail | Fail | 0.83 ± 0.41 | 0.73 | 64.7 ± 25.4 | 48.5 |
|  | Pass | 1.00 ± 0.02 | 0.11 | 93.9 ± 3.9 | 13.0 |

**Supplementary Table 2.** Mean Values, Standard Deviations (SD), and Ranges of TLD-to-TPS Dose Ratios and Gamma Passing Rates across Treatment Parameters for the Proton H&N Phantom.

|  |  | Average PTV TLD | | Average  Parotid TLD | | Spinal Cord TLD | | Average  Gamma (%) | |
| --- | --- | --- | --- | --- | --- | --- | --- | --- | --- |
|  | **Category** | Mean ± SD | Range | Mean ± SD | Range | Mean ± SD | Range | Mean ± SD | Range |
| Machine | Hitachi | 0.98 ± 0.01 | 0.04 | 1.01 ± 0.08 | 0.27 | 1.01 ± 0.11 | 0.37 | 96.2 ± 2.3 | 7.0 |
|  | IBA | 0.98 ± 0.02 | 0.09 | 1.05 ± 0.13 | 0.57 | 0.96 ± 0.11 | 0.46 | 94.6 ± 3.2 | 12.0 |
|  | Mevion | 0.98 ± 0.04 | 0.13 | 1.00 ± 0.04 | 0.14 | 1.02 ± 0.13 | 0.43 | 90.7 ± 6.6 | 23.5 |
|  | Varian | 0.97 ± 0.02 | 0.06 | 1.00 ± 0.07 | 0.32 | 1.01 ± 0.05 | 0.19 | 93.4 ± 2.3 | 9.0 |
|  | Other | 0.96 ± 0.03 | 0.09 | 1.02 ± 0.08 | 0.22 | 1.04 ± 0.13 | 0.36 | 92.8 ± 3.0 | 8.5 |
| Technique | PBS | 0.98 ± 0.02 | 0.13 | 1.03 ± 0.1 | 0.72 | 1.02 ± 0.08 | 0.40 | 94.1 ± 3.0 | 14.0 |
|  | Passive Scatter | 0.96 ± 0.03 | 0.09 | 0.98 ± 0.02 | 0.08 | 0.98 ± 0.15 | 0.52 | 90.9 ± 7.5 | 21.5 |
|  | Uniform Scanning | 0.97 | - | 1.00 | - | 0.65 | - | 91.5 | - |
| TPS | Eclipse | 0.98 ± 0.02 | 0.09 | 0.99 ± 0.08 | 0.41 | 1.02 ± 0.06 | 0.25 | 93.3 ± 2.8 | 11.5 |
|  | RayStation | 0.98 ± 0.03 | 0.13 | 1.04 ± 0.1 | 0.58 | 1.01 ± 0.12 | 0.64 | 94.4 ± 3.1 | 14.0 |
| Algorithm | Monte Carlo | 0.98 ± 0.03 | 0.12 | 1.04 ± 0.11 | 0.58 | 1.03 ± 0.09 | 0.40 | 94.1 ± 3.3 | 14.0 |
|  | Pencil Beam | 0.97 ± 0.03 | 0.11 | 1.00 ± 0.07 | 0.41 | 0.99 ± 0.11 | 0.64 | 93.3 ± 4.5 | 21.5 |
| Pass / Fail | Fail | 0.93 ± 0.02 | 0.03 | 1.02 ± 0.08 | 0.22 | 0.93 ± 0.13 | 0.30 | 85.9 ± 6.3 | 14.5 |
|  | Pass | 0.98 ± 0.02 | 0.12 | 1.02 ± 0.09 | 0.72 | 1.01 ± 0.10 | 0.64 | 94.3 ± 2.9 | 14.0 |

**Supplementary Table 3.** Mean Values, Standard Deviations (SD), and Ranges of TLD-to-TPS Dose Ratios and Gamma Passing Rates across Treatment Parameters for the Proton Spine Phantom.

|  |  | Average PTV TLD | | Sagittal Gamma (%) | | Coronal Gamma (%) | |
| --- | --- | --- | --- | --- | --- | --- | --- |
|  | **Category** | Mean ± SD | Range | Mean ± SD | Range | Mean ± SD | Range |
| Machine | Hitachi | 0.99 + 0.01 | 0.02 | 85.0 ± 12.1 | 30.0 | 96.0 ± 3.8 | 10.0 |
|  | IBA | 0.99 + 0.02 | 0.06 | 87.9 ± 4.9 | 15.0 | 93.2 ± 3.1 | 10.0 |
|  | Mevion | 0.99 + 0.02 | 0.06 | 90.7 ± 5.0 | 12.0 | 93.7 ± 2.3 | 7.0 |
|  | Varian | 0.99 + 0.02 | 0.06 | 86.9 ± 7.0 | 23.0 | 91.0 ± 4.1 | 13.0 |
|  | Other | 0.96 + 0.02 | 0.03 | - | - | - | - |
| Technique | PBS | 0.99 + 0.02 | 0.07 | 87.4 ± 7.3 | 32.0 | 93.2 ± 3.8 | 17.0 |
|  | Passive Scatter | 0.98 + 0.02 | 0.07 | 89.6 ± 5.4 | 12.0 | 93.4 ± 2.7 | 7.0 |
|  | Uniform Scanning | 0.98 + 0.02 | 0.04 | - | - | - | - |
| TPS | Eclipse | 0.99 + 0.02 | 0.07 | 86.2 ± 9.0 | 32.0 | 92.8 ± 4.3 | 15.0 |
|  | Raystation | 1.00 + 0.02 | 0.07 | 89.1 ± 4.9 | 17.0 | 93.9 ± 3.2 | 11.0 |
|  | XiO | 0.98 + 0.02 | 0.04 | - | - | - | - |
| Algorithm | Monte Carlo | 1.00 ± 0.02 | 0.07 | 88.4 ± 5.4 | 17.0 | 94.1 ± 3.2 | 11.0 |
|  | Pencil Beam | 0.99 ± 0.02 | 0.08 | 87.4 ± 8.0 | 32.0 | 92.8 ± 3.8 | 15.0 |
| Pass / Fail | Fail | 1.01 + 0.02 | 0.04 | 74.2 ± 8.0 | 18.0 | 91.2 ± 6.6 | 14.0 |
|  | Pass | 0.99 + 0.02 | 0.08 | 90.0 ± 3.7 | 12.0 | 93.5 ± 3.0 | 12.0 |

**Supplementary Table 4.** Mean Values, Standard Deviations (SD), and Ranges of TLD-to-TPS Dose Ratios and Gamma Passing Rates across Treatment Parameters for the Proton Prostate Phantom.

|  |  | Average PTV TLD | | Average Femoral TLD | | Average Gamma (%) | |
| --- | --- | --- | --- | --- | --- | --- | --- |
|  | **Category** | Mean ± SD | Range | Mean ± SD | Range | Mean ± SD | Range |
| Machine | Hitachi | 0.98 ± 0.02 | 0.08 | 0.97 ± 0.03 | 0.11 | 92.0 ± 8.6 | 29.0 |
|  | IBA | 0.99 ± 0.03 | 0.13 | 0.97 ± 0.03 | 0.16 | 90.4 ± 9.3 | 34.5 |
|  | Mevion | 1.00 ± 0.03 | 0.12 | 0.99 ± 0.03 | 0.09 | 88.9 ± 10.2 | 40.0 |
|  | Varian | 1.00 ± 0.02 | 0.07 | 0.97 ± 0.03 | 0.12 | 92.5 ± 6.7 | 21.0 |
| Technique | PBS | 1.00 ± 0.03 | 0.12 | 0.97 ± 0.03 | 0.15 | 91.2 ± 8.3 | 40.5 |
|  | Passive Scatter | 0.98 ± 0.03 | 0.11 | 0.98 ± 0.03 | 0.10 | 90.9 ± 8.8 | 25.5 |
|  | Uniform Scanning | 0.95 ± 0.02 | 0.06 | 0.95 ± 0.03 | 0.07 | 87.9 ± 12 | 33.5 |
| TPS | Eclipse | 0.98 ± 0.03 | 0.10 | 0.96 ± 0.03 | 0.14 | 92.6 ± 7.8 | 34.5 |
|  | RayStation | 1.00 ± 0.03 | 0.12 | 0.98 ± 0.03 | 0.13 | 89.8 ± 9.6 | 40.5 |
|  | XiO | 0.97 ± 0.04 | 0.13 | 0.97 ± 0.04 | 0.14 | 88.8 ± 11 | 27.5 |
| Algorithm | Monte Carlo | 1.00 ± 0.02 | 0.12 | 0.98 ± 0.03 | 0.11 | 88.6 ± 10 | 40.0 |
|  | Pencil Beam | 0.98 ± 0.03 | 0.13 | 0.96 ± 0.03 | 0.16 | 91.6 ± 8.3 | 34.5 |
| Pass / Fail | Fail | 0.98 ± 0.04 | 0.13 | 0.98 ± 0.03 | 0.14 | 77.6 ± 7.7 | 29.0 |
|  | Pass | 0.99 ± 0.03 | 0.13 | 0.97 ± 0.03 | 0.15 | 94.8 ± 3.7 | 13.0 |

**Supplementary Table 5.** Mean Values, Standard Deviations (SD), and Ranges of TLD-to-TPS Dose Ratios and Gamma Passing Rates across Treatment Parameters for the Proton Lung Phantom.

|  |  | Average PTV TLD | | Sagittal Gamma (%) | | Coronal Gamma (%) | | Axial Gamma (%) | |
| --- | --- | --- | --- | --- | --- | --- | --- | --- | --- |
|  | **Category** | Mean ± SD | Range | Mean ± SD | Range | Mean ± SD | Range | Mean ± SD | Range |
| Machine | Hitachi | 0.98 ± 0.02 | 0.08 | 88.8 ± 13.8 | 45.0 | 89.7 ± 12.4 | 43.0 | 88.9 ± 5.9 | 21.0 |
|  | IBA | 0.97 ± 0.03 | 0.16 | 86.9 ± 9.6 | 36.0 | 88.8 ± 10.3 | 43.0 | 86.1 ± 13.7 | 54.0 |
|  | Mevion | 0.98 ± 0.02 | 0.09 | 87.8 ± 10.5 | 43.0 | 86.5 ± 10.5 | 35.0 | 81.7 ± 13.3 | 44.0 |
|  | Varian | 0.97 ± 0.03 | 0.14 | 85.8 ± 11.6 | 41.0 | 88.1 ± 9.2 | 40.0 | 83.3 ± 14.0 | 55.0 |
|  | Other | 0.97 ± 0.04 | 0.13 | 82.4 ± 11.8 | 34.0 | 85.0 ± 8.8 | 25.0 | 85.9 ± 6.3 | 19.0 |
| Technique | IMPT | 0.97 ± 0.03 | 0.16 | 87.1 ± 11.2 | 45.0 | 88.2 ± 10.6 | 43.0 | 84.4 ± 12.9 | 55.0 |
|  | Passive Scatter | 0.97 ± 0.03 | 0.09 | 84.9 ± 10.7 | 34.0 | 85.5 ± 9.3 | 29.0 | 84.7 ± 12.8 | 41.0 |
|  | Uniform Scanning | 0.96 ± 0.01 | 0.04 | 85.0 ± 6.7 | 19.0 | 89.3 ± 3.7 | 10.0 | 87.3 ± 9.9 | 25.0 |
| TPS | Eclipse | 0.97 ± 0.03 | 0.14 | 85.7 ± 10.6 | 42.0 | 87.8 ± 9.1 | 40.0 | 81.9 ± 12.9 | 56.0 |
|  | RayStation | 0.98 ± 0.03 | 0.16 | 88.2 ± 11.6 | 45.0 | 88.1 ± 11.9 | 43.0 | 86.3 ± 13.4 | 53.0 |
|  | XiO | 0.96 ± 0.01 | 0.04 | 84.8 ± 7.5 | 19.0 | 89.8 ± 3.9 | 10.0 | 86.6 ± 10.9 | 25.0 |
|  | Other | 0.99 ± 0.03 | 0.09 | 84.8 ± 10.9 | 34.0 | 86.0 ± 7.8 | 25.0 | 87.8 ± 5.2 | 17.0 |
| Algorithm | Monte Carlo | 0.98 ± 0.03 | 0.16 | 87.7 ± 11.6 | 45.0 | 89.0 ± 10.5 | 43.0 | 86.3 ± 13.2 | 53.0 |
|  | Pencil Beam | 0.97 ± 0.03 | 0.13 | 85.6 ± 10.0 | 42.0 | 86.7 ± 9.7 | 42.0 | 83.6 ± 12.0 | 56.0 |
| Motion | Breath Hold | 0.97 ± 0.03 | 0.11 | 86.8 ± 12.6 | 38.0 | 89.9 ± 11.6 | 36.0 | 82.4 ± 13.1 | 51.0 |
|  | Gating | 0.99 ± 0.01 | 0.02 | 94.0 ± 3.5 | 10.0 | 92.8 ± 6.5 | 19.0 | 84.0 ± 5.6 | 15.0 |
|  | ITV | 0.97 ± 0.03 | 0.16 | 86.0 ± 10.2 | 44.0 | 86.8 ± 9.4 | 42.0 | 85.5 ± 12.1 | 45.0 |
|  | Static | 0.98 ± 0.04 | 0.1 | 85.8 ± 16.9 | 40.0 | 88.2 ± 15.9 | 37.0 | 84.2 ± 22.8 | 54.0 |
| Pass / Fail | Fail | 0.96 ± 0.04 | 0.16 | 77.2 ± 11.8 | 40.0 | 80.7 ± 12.0 | 43.0 | 73.1 ± 12.6 | 51.0 |
|  | Pass | 0.98 ± 0.02 | 0.11 | 92.4 ± 4.5 | 16.0 | 92.2 ± 5.4 | 20.0 | 91.6 ± 5.7 | 20.0 |

**Supplementary Table 6.** Mean Values, Standard Deviations (SD), and Ranges of TLD-to-TPS Dose Ratios and Gamma Passing Rates across Treatment Parameters for the Proton Liver Phantom.

|  |  | **Average TLD PTV1** | | **Average Gamma**  **PTV1 (%)** | | **Average TLD PTV2** | | **Average Gamma**  **PTV2 (%)** | |
| --- | --- | --- | --- | --- | --- | --- | --- | --- | --- |
|  | **Category** | Mean ± SD | Range | Mean ± SD | Range | Mean ± SD | Range | Mean ± SD | Range |
| **Machine** | Hitachi | 0.97 ± 0.02 | 0.06 | 95.1 ± 1.9 | 5.0 | 0.97 ± 0.01 | 0.03 | 90.4 ± 7.6 | 18.0 |
|  | IBA | 0.97 ± 0.02 | 0.10 | 86.6 ± 14.5 | 55.0 | 0.97 ± 0.02 | 0.08 | 87.2 ± 11.8 | 48.0 |
|  | Mevion | 0.96 ± 0.04 | 0.15 | 77.0 ± 16.7 | 49.0 | 0.97 ± 0.03 | 0.12 | 79.6 ± 14.0 | 50.0 |
|  | Varian | 0.98 ± 0.02 | 0.07 | 91.0 ± 5.7 | 19.0 | 0.98 ± 0.02 | 0.07 | 89.6 ± 6.9 | 21.0 |
| **Technique** | PBS | 0.97 ± 0.02 | 0.14 | 87.8 ± 12.3 | 55.0 | 0.98 ± 0.02 | 0.11 | 87.7 ± 11.1 | 50.0 |
|  | Passive Scatter | 0.96 ± 0.03 | 0.11 | 77.5 ± 16.2 | 49.0 | 0.96 ± 0.03 | 0.09 | 79.3 ± 11.5 | 42.0 |
|  | Uniform Scanning | 0.95 ± 0.03 | 0.08 | 85.4 ± 17.3 | 48.5 | 0.96 ± 0.02 | 0.06 | 82.9 ± 15.2 | 44.0 |
| **TPS** | Eclipse | 0.96 ± 0.03 | 0.10 | 81.8 ± 15.0 | 50.0 | 0.96 ± 0.02 | 0.08 | 83.4 ± 11.8 | 44.0 |
|  | Raystation | 0.98 ± 0.02 | 0.12 | 87.5 ± 12.9 | 54.5 | 0.98 ± 0.02 | 0.11 | 86.4 ± 13.4 | 50.0 |
|  | XiO | 0.96 ± 0.01 | 0.03 | 91.6 ± 6.7 | 18.5 | 0.96 ± 0.01 | 0.03 | 90.6 ± 5.5 | 14.0 |
| **Algorithm** | Monte Carlo | 0.98 ± 0.03 | 0.13 | 88.1 ± 13.1 | 52.5 | 0.98 ± 0.02 | 0.11 | 86.9 ± 13.6 | 50.0 |
|  | Pencil Beam | 0.96 ± 0.03 | 0.11 | 84.2 ± 14.5 | 55.0 | 0.97 ± 0.02 | 0.09 | 84.9 ± 11.3 | 44.5 |
| **Motion** | Breath Hold | 0.97 ± 0.02 | 0.08 | 91.7 ± 7.0 | 23.5 | 0.97 ± 0.02 | 0.06 | 88.8 ± 9.4 | 31.0 |
|  | Gating | 0.97 ± 0.02 | 0.05 | 81.7 ± 15.1 | 36.5 | 0.98 ± 0.03 | 0.07 | 80.8 ± 17.7 | 44.0 |
|  | ITV | 0.97 ± 0.03 | 0.14 | 84.5 ± 15.2 | 55.0 | 0.97 ± 0.02 | 0.12 | 84.9 ± 12.3 | 50.0 |
|  | Static | 0.96 ± 0.05 | 0.11 | 84.1 ± 14.4 | 32.5 | 0.97 ± 0.03 | 0.07 | 88.5 ± 9.6 | 23.0 |
| **Pass / Fail** | Fail | 0.95 ± 0.03 | 0.11 | 75.1 ± 15.7 | 52.0 | 0.96 ± 0.02 | 0.07 | 76.3 ± 12.2 | 47.0 |
|  | Pass | 0.98 ± 0.02 | 0.11 | 93.6 ± 3.8 | 14.0 | 0.98 ± 0.02 | 0.10 | 92.74 ± 4.7 | 21.0 |
